# Supplementary material for: Wood Anatomical and Demographic Similarities Between Self-Standing Liana and Tree Seedlings in Tropical Dry Forests of Colombia
Source: Plants (Basel). 2024 Oct 29;13(21):3023. doi: 10.3390/plants13213023 (PMC11548046; doi:10.3390/plants13213023)
Supplement: Supplementary file 1 [file plants-13-03023-s001.zip › plants-3130135-supplementary.pdf]

SUPPORTING INFORMATION

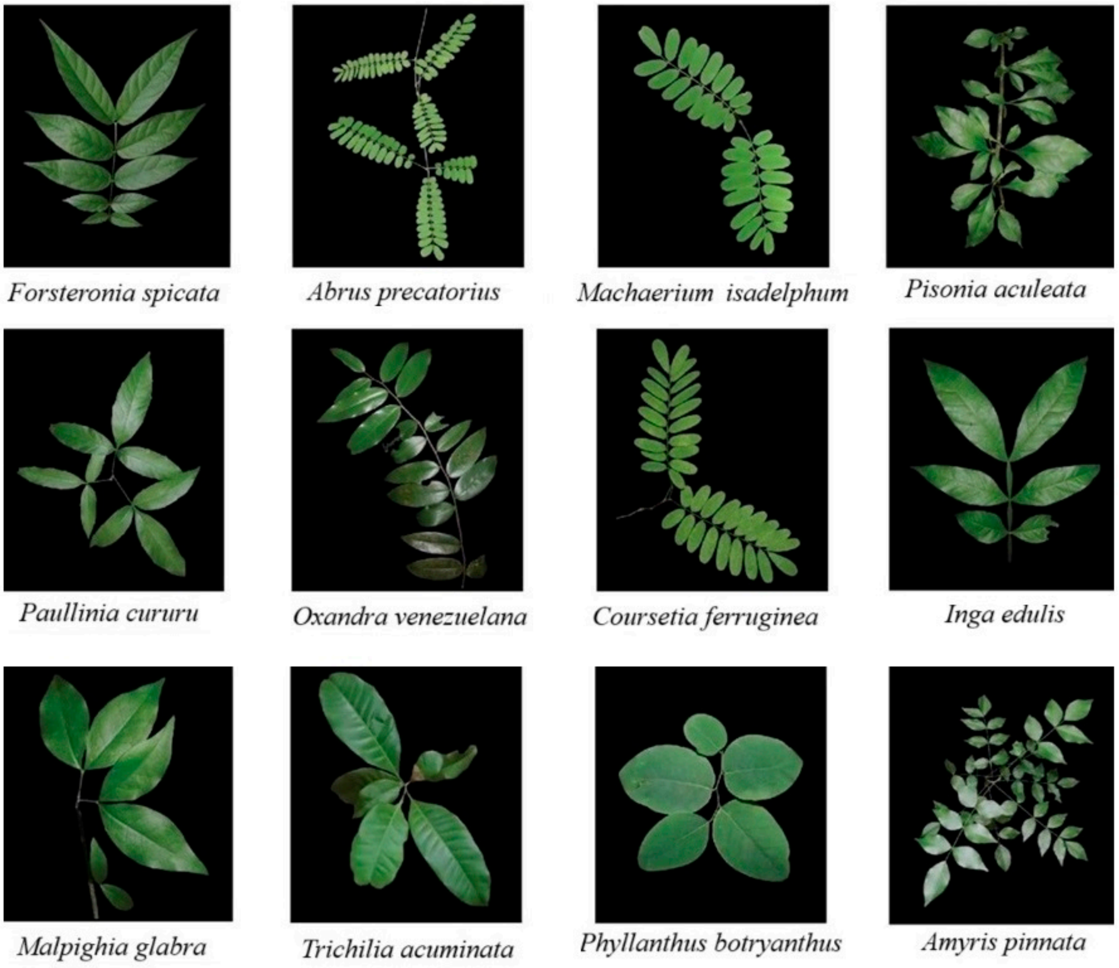

Figure S1. Epecies selected for analysis. See Table 4 for more information.

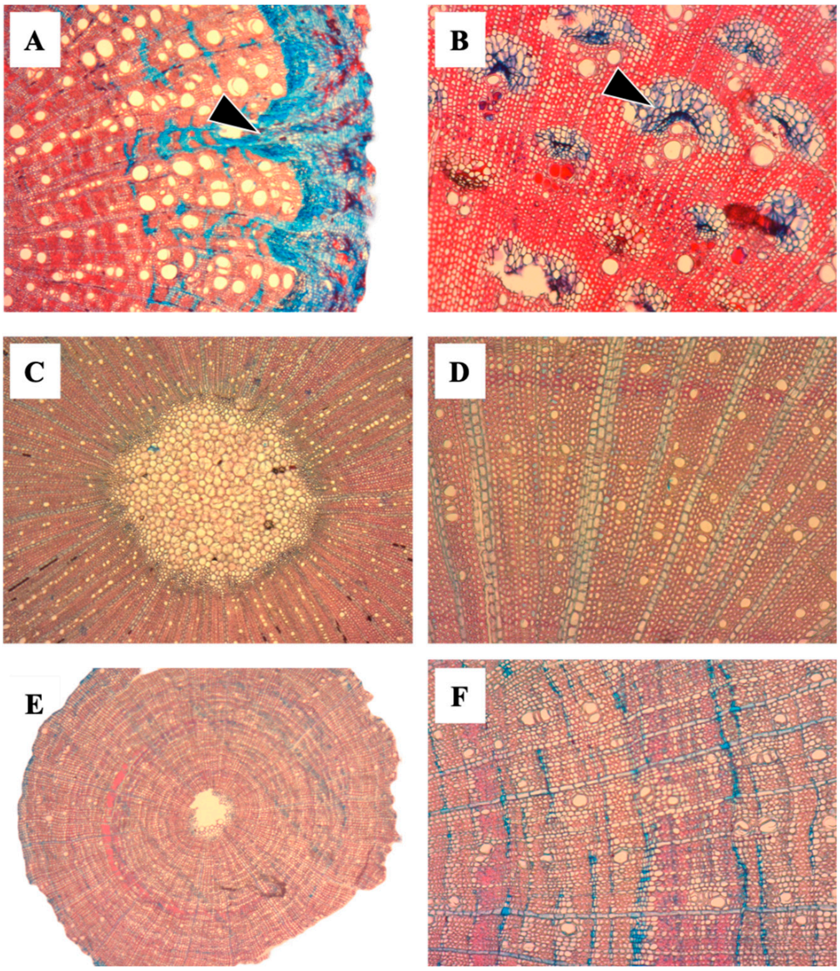

Figure S2. Anatomical images of four liana seedling species. A) Phloem edges (black head arrow) in *Abrus precatorius*; B) groups of internal phloem (black head arrow) in *Pisonia aculeata*; C-D) cross-sections of *Bignonia pterocalyx* with no phloem wedges typical of Bignoniaceae lianas; E-F) *Machaerium isadelphum*: with a rounded stem and no successive cambia respectively.

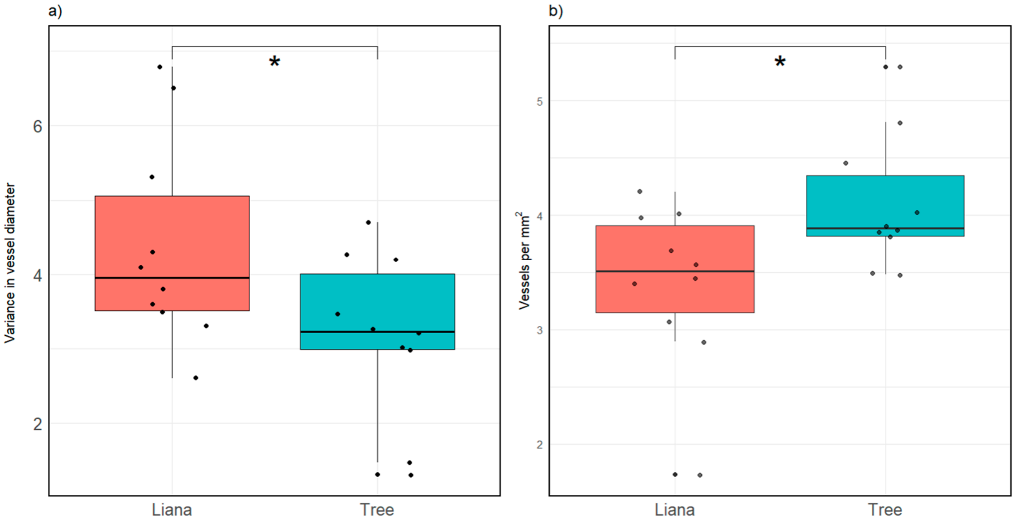

Figure S3. t-test comparison of a) variance in vessel diameter and b) vessel density. * = significance (alpha = 0.05).

Table S1. Summary of wood traits measured on seedlings of the 20 species selected. Mean, standard deviation, and range are shown.

Trait	mean	sd	range
Axial parenchyma fraction	24.39	15.2	0.45 - 53.92
Mean hydraulically weighted vessel diameter	47.28	23.97	16.8 - 143.89
Horizontal pit membrane diameter aperture	7.51	2.03	4.51 - 13.14
Fiber fraction	5.1	16.08	17.88 - 85.44
Fiber wall thickness	3.68	1.36	0 - 8.89
Radial parenchyma fraction	14.84	9.02	3.26 - 47.2
Total parenchyma fraction	38.04	15.61	5.37 - 72.21
Variance in vessel diameter	121.22	231.13	3.72 - 885.35
Vessel clustering index	1.38	0.89	0.25 - 5.56
Vessel density	59.93	46.18	3.05 - 238.48
Vessel fraction	7.86	5.59	2.59 - 36.33
Wood density	0.56	0.11	0.02 - 0.81
